# Adaptação transcultural e evidências de validade psicométricas da *Family Health Scale* para o português brasileiro

**DOI:** 10.1590/0102-311XPT048823

**Published:** 2023-12-08

**Authors:** Evanizia Pinheiro de Oliveira, José Cláudio Garcia Lira, Ivana Cristina de Holanda Cunha Barreto, Ana Cristina Pereira de Jesus Costa, Roberto Wagner Freire de Freitas, Danilo Ferreira de Sousa, Márcio Flávio Moura de Araújo

**Affiliations:** 1 Fundação Oswaldo Cruz, Eusébio, Brasil.; 2 Universidade Federal do Maranhão, Imperatriz, Brasil.; 3 Universidade Federal do Ceará, Fortaleza, Brasil.

**Keywords:** Psicometria, Atenção Primária à Saúde, Saúde da Família, Estudo de Validação, Psychometrics, Primary Health Care, Family Health, Validation Study, Psicometría, Atención Primaria de Salud, Salud de la Familia, Estudio de Validación

## Abstract

Os objetivos deste estudo foram realizar a tradução e adaptação transcultural da *Family Health Scale* (*Escala de Saúde Familiar*) para a língua portuguesa brasileira e analisar evidências de validade psicométricas dessa escala. Os 32 itens sobre a saúde familiar foram adaptados transculturalmente. Para a mensuração das evidências de validade do conteúdo, utilizou-se o cálculo do índice de validade de conteúdo das características semântica, idiomática, cultural e conceitual de cada item e da escala. Um pré-teste para identificação de evidência de validade foi realizado com 40 famílias. Em outro momento, a aplicação do instrumento foi executada com 354 famílias, em uma cidade no Nordeste do Brasil. O índice de concordância entre os juízes variou de 0,84, para os itens da escala, a 0,98, para a escala total, conforme o coeficiente de Kendall. As evidências de validade psicométricas mostram-se adequadas, conforme alfa de Cronbach. A maior parte das famílias teve um grau de saúde moderado, conforme aplicação da escala. Assim, a *Family Health Scale*, versão brasileira, apresentou equivalência conceitual, semântica, cultural e operacional em relação aos itens originais e propriedades psicométricas satisfatórias para a aplicação direcionada à população brasileira, atestando eficácia e segurança de sua utilização.

## Abstract (en)

This study aimed to translate and cross-culturally adapt the *Family Health Scale* into Brazilian Portuguese and analyze evidence of its psychometric validity. The 32 items on family health were cross-culturally adapted, using the content validity index to calculate semantic, idiomatic, cultural, and conceptual characteristics of the scale and its items. A pre-test to identify evidence of validity was applied to 40 families. At another time, the instrument was applied to 354 families in a Brazilian northeastern city. The index of agreement between the raters ranged from 0.84 for the scale items to 0.98 for the total scale, according to Kendall’s coefficient. According to Cronbach’s alpha, evidence of psychometric validity is adequate. Most families had a moderate degree of health, according to the scale. Therefore, the Brazilian version of the *Family Health Scale* showed conceptual, semantic, cultural, and operational equivalence with the original items, along with satisfactory psychometric properties for use among the Brazilian population with effectiveness and safety.

## Resumen

El objetivo de este estudio fue traducir y realizar la adaptación intercultural de *Family Health Scale* (*Escala de Salud Familiar*) al portugués brasileño, y analizar la evidencia de la validez psicométrica de esta escala. Los 32 ítems sobre salud familiar pasaron por una adaptación transcultural y, para medir la evidencia de validez de contenido, se utilizó el cálculo del índice de validez de contenido de las características semántica, idiomática, cultural y conceptual de cada ítem, y la escala. Se realizó una prueba previa con 40 familias para identificar la evidencia de validez. En otro momento, el instrumento se aplicó a 354 familias en una ciudad del Nordeste de Brasil. El índice de acuerdo entre los jueces osciló entre 0,84 para los ítems de la escala y 0,98 para la escala total de acuerdo con el coeficiente de Kendall. La evidencia de validez psicométrica es adecuada conforme el alfa de Cronbach. La mayoría de las familias tenían un grado de salud moderado de acuerdo con la aplicación de la escala. Por lo tanto, la *Family Health Scale*, versión brasileña, presentó equivalencia conceptual, semántica, cultural y operativa con relación a los ítems originales, y propiedades psicométricas satisfactorias para la aplicación en la población brasileña, lo que da fe de la eficacia y seguridad de su uso.

## Introdução

A adoção de uma abordagem proativa na promoção da saúde e prevenção de doenças e agravos é necessária devido, principalmente, ao aumento global da prevalência de doenças crônicas não transmissíveis ^1^. Parte disso é influenciada pelas condições estruturais, sociais e culturais que sustentam o bem-estar e precisam estar presentes para o alcance de uma saúde efetiva ^2^. Nesse sentido, a família tem lugar central na influência dessas condições, sendo uma das principais contribuintes para o processo saúde-doença de uma pessoa ^3^.

A família pode favorecer, por exemplo, um melhor comportamento de saúde, a adesão ao tratamento, o letramento funcional em saúde, o conforto e o cuidado, dispensados durante a atenção à saúde nos cuidados primários ^2^. O conceito de saúde das famílias, por sua vez, requer o entendimento de como se desenvolve a interseção da saúde de cada membro da família, suas interações e capacidades, bem como os recursos físicos, sociais, emocionais, econômicos e de saúde de uma família ^4^. Logo, compreender sua configuração e organização, considerando suas condições de vida e aspectos psicossociais, direciona profissionais de saúde de cuidados primários a uma melhor assistência, aprimorando o gerenciamento em saúde e o alcance de metas ^2^^,^^4^.

Ainda que haja uma ampla gama de instrumentos que visam identificar necessidades de cuidado à pessoa e a suas famílias na atenção primária à saúde (APS) ^5^^,^^6^^,^^7^, é escasso o uso de instrumentos que consideram pessoas como parte de uma família ou que enfocam a saúde familiar e suas demandas. Isso pode limitar discussões sobre o tema, além do desenvolvimento e da execução de programas e políticas públicas de saúde que visem atender às necessidades das famílias em seus diversos contextos.

Com base nisso, em 2020, foi criada a *Family Health Scale* (*Escala de Saúde Famíliar*), em tradução livre, em conjunto com pesquisadores da Universidade Brigham Young (Provo, Estados Unidos), Universidade de Minnesota (Twin, Estados Unidos) e Universidade de Buffalo (Buffalo, Estados Unidos) ^8^. Até o momento, além do contexto estadunidense, as evidências de validade da escala só foram testadas na população chinesa ^9^. Essa escala tem como objetivo mensurar o nível de saúde das famílias e considera situações de saúde que possam requerer intervenções diferenciadas - e que, sem elas, a saúde familiar estaria em risco. Diferentemente de outros instrumentos disponíveis na literatura, que investigam as condições socioeconômicas e demográficas, essa escala dá ênfase às questões psicossociais e relacionais. Essas características são fundamentais para o suporte aos profissionais da Estratégia Saúde da Família (ESF), que rotineiramente precisam realizar diagnósticos precoces e desenvolver condutas terapêuticas focados na família. No entanto, não há registro de qualquer tradução, adaptação, evidências de validade ou aplicação dessa escala no contexto brasileiro.

Assim, os objetivos deste estudo foram: (1) realizar a tradução e adaptação transcultural da *Family Health Scale* para o Brasil; e (2) analisar evidências de validade psicométricas da *Family Health Scale* (versão brasileira) com famílias acompanhadas pela ESF.

## Métodos

### Desenho do estudo

Este é um estudo metodológico, com delineamento transversal e abordagem quantitativa, de tradução e adaptação transcultural da *Family Health Scale* para o contexto brasileiro.

### Instrumento

A *Family Health Scale*^8^ é um instrumento que permite compreender as tendências de saúde de um grupo familiar e a interseção entre a saúde individual, familiar e comunitária. Essa escala tem 32 itens na sua forma longa multifatorial e 10 itens em sua forma curta uniforme, sendo ambas aplicáveis às famílias. Nelas, são abordados quatro fatores: (1) processo de saúde social e emocional da família; (2) estilo de vida saudável para a família; (3) recursos de saúde da família; e (4) suportes sociais externos da família. Cada um desses fatores tem vários questionamentos a serem aplicados à família, que conduzirão a um diagnóstico e a um posterior planejamento de intervenções.

As opções de resposta em todos os itens estão em uma escala Likert de 5 pontos, sendo que as respostas pontuadas de 1 a 3 recebem 0 ponto e as assinaladas em 4 ou 5 recebem 1 ponto. De modo geral, os itens a serem assinalados na escala variam de “discordo totalmente” a “concordo totalmente”. Entretanto, os itens 1, 5, 20-24, 29-32 da escala longa pontuam de forma reversa, ou seja, itens marcados de 1 a 3 passam a valer 1 ponto; e aqueles selecionados em 4 ou 5, 0 ponto. Da mesma forma, os itens 23, 31 e 32 do questionário curto sofrem tal reversão. Após o compilado de respostas assinaladas, chega-se à classificação da saúde da família, adotando como base a escala curta: saúde ruim = 0-5 pontos; saúde familiar moderada = 6-8 pontos; saúde familiar excelente = 9-10 pontos ^8^.

### Etapas da tradução, adaptação transcultural e pré-teste

**Figura 1:**
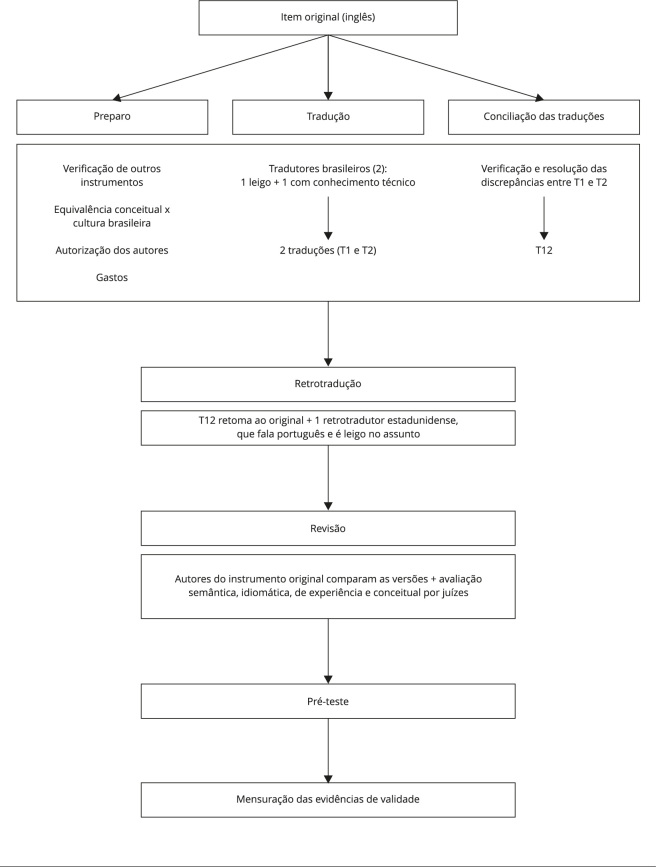
Fluxograma do processo de adaptação transcultural e validação da *Family Health Scale* (*Escala de Saúde Familiar*).

Em nosso estudo, seguimos o percurso metodológico composto pelas seguintes etapas: (a) preparo; (b) tradução; (c) conciliação das traduções; (d) retrotradução; (e) revisão; (f) pré-teste; e (g) validação ^10^ (Figura 1).

Fonte: elaboração própria.

Durante a etapa de preparo do estudo, a verificação da existência de outros instrumentos validados no Brasil que aferiram os mesmos desfechos foi realizada por buscas nas bases e bibliotecas virtuais PubMed, SciELO, BIREME e na literatura cinza. Em seguida, foi realizada a equivalência conceitual entre a escala e os valores culturais da população-alvo. Ademais, foi solicitada autorização para adaptação transcultural à equipe que construiu o instrumento original. Por fim, foram avaliados o cronograma e os custos envolvidos para cumprir essas etapas.

A etapa de tradução do instrumento original para o português brasileiro foi realizada por dois tradutores nativos do Brasil, fluentes na língua inglesa e residentes no país. Um dos tradutores era leigo na área e construiu a tradução T1. Já a tradução T2 foi desenvolvida por outro tradutor, com doutorado em Enfermagem e especialização na área. Na sequência, foram realizadas comparações entre elas, eliminando discrepâncias e assegurando uma semântica adequada e clara para a compreensão do instrumento.

Após isso, foi feita a retrotradução, também chamada de versão T12. Para tal, um tradutor estrangeiro, que tinha a língua inglesa como nativa e era fluente em português brasileiro, sem conhecimento do assunto, desenvolveu essa etapa. Em seguida, duas revisões foram realizadas. Primeiro, pelos autores da versão original do instrumento, que fizeram uma comparação da T12 com a versão original, constatando a equivalência e a qualidade. Em seguida, foi constituído um comitê de juízes multidisciplinar. Para selecionar a quantidade ideal de juízes, a literatura foi consultada ^11^^,^^12^, chegando-se ao número de oito juízes, todos da área da saúde. Eles tinham doutorado, conhecimento sobre o assunto, experiência em saúde pública e declararam ter domínio da língua inglesa. Cada um dos juízes recebeu quatro documentos por e-mail: o termo de consentimento livre e esclarecido; a versão original da escala, em inglês; a síntese das traduções T12; e a retrotradução. Os juízes então avaliaram a síntese das traduções T12, isto é, a retrotradução. Para registro de suas sugestões, foi disponibilizado um formulário eletrônico.

Por fim, a etapa de pré-teste consistiu na aplicação da escala traduzida e da versão adaptada ao Brasil, a fim de verificar se esta conseguiu extrair do respondente o quadro de saúde da sua família. Para tanto, essa fase do estudo foi realizada em uma unidade básica de saúde (UBS), localizada na cidade de Fortaleza (Ceará).

Para a seleção da amostra, foi utilizada uma ferramenta virtual, que sorteou nomes de sete ruas adscritas pela UBS. Após isso, os pesquisadores decidiram, por conveniência, que apenas uma numeração de cada rua seria contemplada. No total, 40 residências foram elencadas. No entanto, para participar do estudo, as pessoas deveriam: ter idade entre 18 e 69 anos, com pelo menos cinco anos de estudo e gozando de boa saúde mental. Foram excluídos da pesquisa aqueles que habitualmente não atendem às visitas dos agentes comunitários de saúde, pessoas que estavam em áreas em situação de perigo e aqueles que não estavam mais residindo no endereço selecionado. Quando isso acontecia, uma outra casa era listada. Em seguida, deu-se início à coleta de dados.

### Análise de dados

Com base nos instrumentos aplicados junto aos juízes (evidências de validade do conteúdo) e às famílias (versão traduzida e adaptada), elaboramos um banco de dados com dupla entrada no Excel (https://products.office.com/). Para a mensuração das evidências de validade do conteúdo, optamos pelo cálculo do índice de validade de conteúdo (IVC) das características semântica, idiomática, cultural e conceitual de cada item (I-IVC) e da escala (E-IVC). Consideramos o valor mínimo de 0,8 para o I-IVC de itens e E-IVC na avaliação geral do instrumento ^13^^,^^14^.

Para avaliar o grau de concordância entre os juízes, empregamos o coeficiente de concordância de Kendall, uma vez que é capaz de medir os graus de associação entre especialistas na classificação de variáveis qualitativas ordinais com ≥ 3 níveis. Ademais, avaliamos o resultado desse coeficiente, sendo: < 0,2 (concordância ruim); 0,21-0,4 (concordância regular); 0,41-0,6 (concordância moderada); 0,61-0,8 (concordância boa); 0,81-1,0 (concordância muito boa) ^15^. Essa análise foi realizada no software SPSS versão 20.0 (https://www.ibm.com/), com intervalo de 95% de confiança (IC95%).

### Evidências de validade psicométrica

Para mensuração das evidências de validade da *Family Health Scale*, um enfermeiro da ESF e mais quatro agentes comunitários de saúde realizaram visitas domiciliares a 354 famílias da cidade de Fortaleza. A amostra foi calculada conforme o número de itens da escala, multiplicado por 1.010. A seleção das famílias aconteceu por meio de um sorteio, realizado por uma ferramenta virtual. Nomes de ruas adscritos pela UBS em que trabalhavam o enfermeiro e os agentes comunitários de saúde foram utilizados. Nessa ocasião, os pesquisadores aplicaram as versões curta e longa da escala, anteriormente submetida ao pré-teste com 40 famílias. Para participar do estudo, as pessoas deveriam: ter idade entre 18 e 69 anos, com pelo menos cinco anos de estudo e gozando de boa saúde mental.

Analisamos a avaliação da estrutura interna e a aplicabilidade do instrumento por meio da análise fatorial exploratória (AFE) e da análise fatorial confirmatória (AFC), após a avaliação dos especialistas. A verificação da adequação da amostra à análise fatorial foi avaliada pelo teste de Kaiser-Meyer-Olkin (KMO), adotando-se valor maior que 0,60 como critério de adequação de ajuste do modelo e teste de esfericidade de Bartlett (p < 0,001).

Utilizou-se mínimo quadrado robusto com peso diagonal (RDWLS) e a correlação policórica, com rotação *Promin* robusta para extração dos fatores. A confiabilidade foi avaliada pelo coeficiente *alfa* (α) de Cronbach, correlação item-total e o estimador ômega (ω) de McDonald. Os softwares utilizados na análise foram o JASP 0.17 (https://jasp-stats.org/previous-versions/) e o JAMOVI versão 1.6 (https://www.jamovi.org/download.html).

### Aspectos éticos

O trabalho foi aprovado pelo Comitê de Ética e Pesquisa com Seres Humanos da Universidade Federal do Ceará (parecer nº 5.418.800/2022).

## Resultados

### Tradução e avaliação da equivalência semântica idiomática, cultural e conceitual

O processo de tradução (T1 e T2) gerou resultados muito próximos, não comprometendo as equivalências relevantes da escala. Após isso, no processo de retrotradução, verificamos que a versão traduzida para o inglês se manteve muito fiel à versão original do instrumento, sendo esta revisada e aprovada pelos próprios criadores da escala original. Por sua vez, na análise de conteúdo dos itens (equivalências semântica, idiomática, cultural e conceitual), realizada por juízes, identificou-se que apenas em sete deles não houve resultado igual a 1 (item 7, 8, 15, 17, 18, 24 e 35). Já o cálculo de E-IVC verificado foi de 0,98. Assim, com base na análise dos juízes, observamos que o grau de concordância entre eles foi elevado (W = 0,98, p < 0,001).

Após a verificação das sugestões dos especialistas, os itens da *Family Health Scale* foram reorganizados e agrupados em quatro fatores: (1) processo de saúde social e emocional da família - itens 1 a 13 (anteriormente itens 1 a 11, 18 e 19), sendo que os itens 18 e 19 foram trocados pelos itens 12 e 13; (2) estilo de vida saudável da família - itens 14 a 19 (anteriormente 12 a 17), sendo que os itens 12 e 13 da versão anterior passaram a ser 18 e 19; (3) apoio social externo à família - itens 20 a 23 (anteriormente 25 a 28); (4) recursos de saúde da família - itens 24 a 32 (anteriormente 20 a 24 e 29 a 32). As mudanças foram realizadas sem que ocorresse perda de equivalência ou alteração na pontuação preestabelecida.

### Pré-teste

A versão traduzida e adaptada ao cenário da ESF foi aplicada junto a 40 famílias brasileiras na cidade de Fortaleza, na etapa do pré-teste. Durante a entrevista para o teste do instrumento, o representante familiar era majoritariamente do gênero feminino (92,5%), com média de idade de 45 anos (desvio padrão - DP ± 12,9) e Ensino Médio completo (60%). As famílias eram compostas, em boa parte, por quatro pessoas (32,5%).

Na aplicação da *Family Health Scale* (versão curta), 82,5% (n = 33) das famílias tiveram saúde familiar “moderada”, seguida de “ruim” (10%, n = 4) e “excelente” (7,5%, n = 3). Já na versão estendida, 52,5% (n = 21), 42,5% (n = 17) e 5% (n = 2) das famílias foram classificadas com saúde familiar “moderada”, “excelente” e “ruim”, respectivamente. A apresentação das versões em inglês e traduções da *Family Health Scale* podem ser conferidas no Material Suplementar (Quadro S1 e Figura S1; https://cadernos.ensp.fiocruz.br/static//arquivo/supl-e00048823_9572.pdf).

### Evidências de validade

Um total de 354 pessoas compôs a amostra e respondeu à versão final da escala. Os participantes foram, predominantemente, mulheres, de cor de pele parda e na faixa etária de 41-65 anos de idade, com média de idade de 44,8 anos (± 15.3). Em 50,6% das famílias entrevistadas, os lares eram coabitados por outros membros da família. O percentual de pessoas casadas ou em união estável foi quase o dobro do percentual de solteiros. Nessas famílias, predominou uma situação economicamente baixa. Em mais da metade das famílias entrevistadas (67,5%), observou-se que a renda familiar mensal para custear as despesas da casa vem de apenas uma pessoa, que geralmente é do gênero masculino (52,3%) (Tabela 1).

**Tabela 1:** Caracterização das famílias entrevistas. Fortaleza, Ceará, Brasil, 2023 (N = 354).

Variável	n (%)
Gênero	
Feminino	284 (81,1)
Masculino	66 (18,9)
Faixa etária (anos)	
Até 20	19 (6,3)
21-40	113 (35,8)
41-65	159 (50,3)
≥ 66	24 (7,7)
Anos de estudo do chefe da família	
Até 5	67 (19,4)
Até 9	88 (25,1)
Até 12	55 (15,7)
Até 15	121 (34,6)
> 15	18 (5,1)
Cor da pele	
Branca	47 (13,6)
Negra	41 (11,8)
Amarela	8 (2,3)
Parda	249 (72,0)
Com quem mora	
Pais	14 (4,0)
Familiares	176 (50,6)
Amigos	1 (0,3)
Companheiro(a)	142 (40,8)
Sozinho	15 (4,3)
Classe econômica	
Média alta	31 (9,1)
Média	153 (43,3)
Baixa	168 (47,6)
Familiares que contribuem com a renda familiar	
Apenas 1	233 (67,5)
2	96 (27,8)
> 2	16 (4,6)

Com base no teste KMO, um pré-requisito para verificação de amostragem adequada, obteve-se valor de 0,808, evidenciando que não é necessário retirar nenhuma questão da versão final do instrumento. Ademais, o teste de esfericidade de Bartlett (p < 0,001) evidenciou que há relação entre as questões. O modelo apresentou índices adequados, não necessitando ajustes em relação às variáveis.

**Figura 2:**
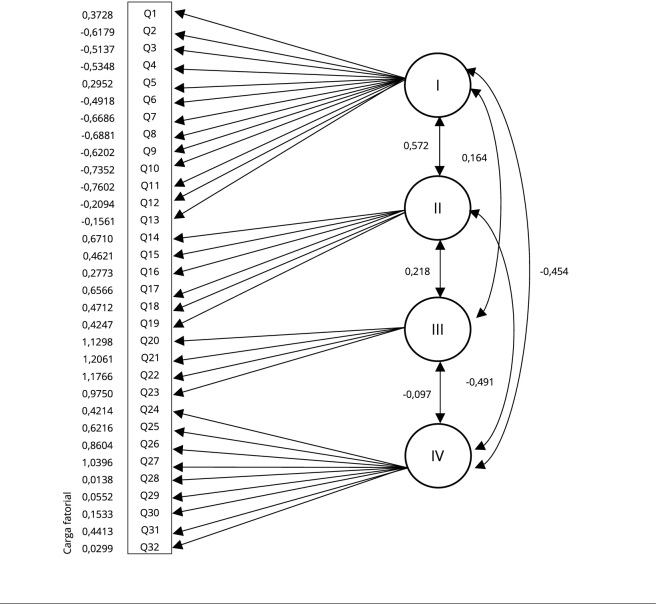
Pictograma da correlação e carga fatorial do modelo final da *Family Health Scale* (*Escala de Saúde Familiar*).

Com a observação do diagrama fatorial, é possível observar as relações entre os fatores: fator I - processo de saúde social e emocional da família; fator II - estilo de vida saudável da família; fator III - apoio social externo à família; e fator IV - recursos de saúde da família. Nesse caso, é importante observar que existe relação relevante entre os fatores I e III; II e IV; e entre os fatores I e IV de forma mais abrangente. Observamos que houve uma relação sistemática entre os fatores da escala, em alguma medida. Entre os fatores I e II, há uma correlação positiva moderada. Por outro lado, os fatores I e IV apresentam uma correlação moderada negativa (Figura 2).

Fonte: elaboração própria. Nota: I - fator processo de saúde social e emocional da família; II - fator estilo de vida saudável; III - fator apoio social externo; IV - fator recursos de saúde.

Os valores identificados pela confiabilidade interna das questões do instrumento foram satisfatórios, conforme demonstrado na Tabela 2.

**Tabela 2:** Indicadores para avaliação das propriedades psicométricas da *Family Health Scale* (*Escala de Saúde Familiar*).

Fatores	<mml:math><mml:mover accent="true"><mml:mrow><mml:mi>X</mml:mi></mml:mrow><mml:mo>-</mml:mo></mml:mover></mml:math>	DP	Correlação item-total	α de Cronbach	ω de McDonald	Carga fatorial
Processo de saúde social e emocional da família						
Q1	1,98	1,512	-0,11259	0,622	0,797	0,3728
Q2	4,62	0,894	0,44787	0,568	0,766	-0,6179
Q3	4,74	0,790	0,35984	0,577	0,770	-0,5137
Q4	4,77	0,692	0,45367	0,574	0,765	-0,5348
Q5	2,70	1,748	-0,02374	0,616	0,793	0,2952
Q6	4,74	0,780	0,42009	0,573	0,765	-0,4918
Q7	4,41	1,203	0,27860	0,577	0,773	-0,6686
Q8	4,54	1,049	0,43279	0,565	0,764	-0,6881
Q9	4,66	0,893	0,38750	0,573	0,766	-0,6202
Q10	4,62	0,909	0,48751	0,565	0,761	-0,7352
Q11	4,55	0,983	0,42606	0,568	0,763	-0,7602
Q12	4,91	0,495	0,31525	0,586	0,771	-0,2094
Q13	4,95	0,321	0,29902	0,590	0,774	-0,1561
Estilo de vida saudável da família						
Q14	4,55	0,947	0,28208	0,580	0,771	0,6710
Q15	4,76	0,633	0,34174	0,582	0,772	0,4621
Q16	4,85	0,532	0,40635	0,581	0,769	0,2773
Q17	4,66	0,721	0,37625	0,578	0,768	0,6566
Q18	4,42	1,122	0,21178	0,584	0,780	0,4712
Q19	4,64	0,863	0,33099	0,578	0,772	0,4247
Apoio social externo à família						
Q20	4,03	1,536	0,21314	0,583	0,785	1,1298
Q21	3,82	1,562	0,25992	0,577	0,783	1,2061
Q22	3,32	1,767	0,17033	0,589	0,785	1,1766
Q23	3,00	1,791	0,12006	0,597	0,787	0,9750
Recursos de saúde da família						
Q24	2,11	1,585	-0,02602	0,614	0,793	0,4214
Q25	2,56	1,657	-0,00490	0,612	0,791	0,6216
Q26	3,01	1,545	0,06019	0,602	0,788	0,8604
Q27	3,35	1,546	0,13735	0,593	0,786	1,0396
Q28	1,37	0,994	-0,02075	0,604	0,794	0,0138
Q29	2,05	1,536	-0,04922	0,615	0,796	0,0552
Q30	2,65	1,731	0,01773	0,611	0,793	0,1533
Q31	3,44	1,756	0,07124	0,603	0,789	0,4413
Q32	1,66	1,279	0,06329	0,599	0,790	0,0299

DP: desvio padrão.

Observamos uma forte covariância negativa entre as variáveis fatoriais I e II, enquanto no comparativo dos fatores II e III, III e IV a relação foi positiva e significativa. As outras estimativas de covariância fatorial seguem um padrão semelhante (Tabela 3).

**Tabela 3:** Covariâncias fatoriais da *Family Health Scale* (*Escala de Saúde Familiar*).

Covariâncias fatoriais		Estimativas	Erro padrão	Z	Valor de p
I	II	-0,6229	0,0427	-14,593	< 0,001
	III	-0,1151	0,0626	-1,838	0,066
	IV	-0,0610	0,0753	-0,811	0,418
II	III	0,1428	0,0655	2,180	0,029
	IV	0,0651	0,0798	0,816	0,414
III	IV	-0,2692	0,0762	-3,533	< 0,001

## Discussão

Este estudo traduziu, adaptou culturalmente e mensurou as evidências de validade psicométricas para o português brasileiro da *Family Health Scale*, um instrumento criado originalmente para avaliar a saúde das famílias estadunidenses. Como parte das etapas de adaptação transcultural e mensuração das evidências de validade, foi realizado um pré-teste, que contou com a participação de 40 famílias acompanhadas pela ESF de uma UBS, corroborando especialistas ^10^^,^^16^ que pontuam que, nessa fase do estudo, a aplicação do instrumento deve atingir de 30 a 40 pessoas membros da população-alvo. Tanto na versão curta quanto na versão longa da escala, os participantes tiveram uma pontuação que enquadrava as famílias com um grau de saúde moderado. Tal dado demonstra semelhança com os valores obtidos na mensuração das evidências de validade da escala original. Além disso, quando encontrado esse nível de classificação (saúde familiar moderada), os autores evidenciaram que há menor chance de depressão grave ou moderada entre os membros da família (p < 0,001) ^8^.

No que se refere aos fatores encontrados na escala, identificamos que há relações entre eles (fatores I e III; fatores II e IV; e fatores I e IV). Esses fatores apontam para contribuições substanciais para a saúde da família e, consequentemente, para os serviços da ESF no Brasil.

Por exemplo, no fator I (processos de saúde social e emocional da família), podemos avaliar as condições de comunicação, segurança emocional, conexão interpessoal, satisfação e enfrentamento no contexto familiar. A literatura mostra que o enfrentamento dos processos de saúde-doença, apoiado pelos membros da família, minimiza desfechos negativos, como sintomas depressivos, e cria ambientes para o desenvolvimento de competências voltadas à resolução de problemas ^17^^,^^18^. Ainda, relações familiares satisfatórias parecem ser fatores de proteção contra os comportamentos de risco que emergem em fases da vida, como na adolescência ^19^. Em contextos sanitários críticos, a resiliência que insurge do ambiente familiar mitiga sentimentos de estresse e melhora a adaptabilidade para o gerenciamento de enfermidades ^20^.

Em nosso estudo, os itens desse fator tiveram associação com aqueles do fator III (apoio social externo à família), que explora as características externas de apoio pessoal e financeiro, e do fator IV (recursos de saúde da família), em que são avaliadas as condições internas e externas de saúde, incluindo padrões individuais de saúde, preocupações familiares, recursos socioeconômicos e eficácia na busca por ajuda. O apoio social pode ser um pilar para promover a saúde dos membros de uma família, ou da própria família como um todo, em distintos estágios da vida, minorando condições de adoecimento, como as ligadas à saúde mental ^21^^,^^22^^,^^23^. Dito isso, é imperioso que profissionais de saúde explorem tais questões e os vínculos de apoio intra e extrafamiliar, a fim de potencializar a compreensão sobre a saúde e o gerenciamento dos casos.

Sobre o acesso aos recursos de saúde e apoios externos, evidências apontam que baixos recursos familiares aumentam as chances de problemas mentais e estresse ^24^^,^^25^. Além disso, a privação de cuidados em saúde, alimentação, vestuário, transporte, moradia e outros determinantes sociais de saúde está atrelada a uma pior saúde física e mental ^26^^,^^27^. Crescer em circunstâncias socioeconômicas desvantajosas pode iniciar uma cadeia de risco ao predispor as pessoas a perfis de comportamento de saúde associados a uma pior qualidade de vida na velhice ^28^. Essas relações projetam um extenso quadro de necessidades dependentes de inúmeros setores sociais e governamentais. No eixo saúde, revela-se uma maior demanda por cuidados de saúde e de expansão de equipes de saúde da família. No Brasil, a ampliação da APS e a dispensação de cuidados propiciados pela ESF são meios significativos para a redução de mortalidade e cenários desfavoráveis de saúde, especialmente nos grupos mais vulneráveis ^29^.

Outro elo dos itens da *Family Health Scale* foi observado entre os fatores II (estilo de vida saudável da família) e IV. O ambiente familiar tem um impacto significativo no desenvolvimento do comportamento alimentar e na prática de atividade física em crianças ^30^, na prevenção de condições crônicas em saúde não transmissíveis nos diferentes ciclos de vida e na promoção de hábitos e estilos de vida saudáveis ^31^. Todavia, comportamentos não saudáveis são identificados em famílias mais pobres ^32^. Um estudo que associou o nível socioeconômico a comportamentos em saúde antes e depois do diagnóstico de condições crônicas mostrou que pessoas com menos recursos financeiros tendem a perpetuar condutas desvantajosas à saúde ^33^. Quando testada com famílias estadunidense, a *Family Health Scale* mostrou que famílias são saudáveis em vários contextos, não dependendo, exclusivamente, de um *status* socioeconômico alto. No entanto, a renda familiar mais alta esteve associada à melhor saúde familiar, provavelmente porque aumenta o acesso aos recursos e diminui a exposição a outros determinantes arrolados ^34^.

A maior preocupação com comportamentos e o apoio a escolhas promissoras têm forte vinculação com as relações familiares, o estímulo entre os membros de uma unidade familiar e os recursos e as condições individuais. Logo, utilizar um instrumento para melhor compreender essa dinâmica contribui para a melhor tomada de decisão em saúde no que diz respeito a intervenções mais precoces e efetivas para cada membro da família, focando o trabalho multi e interdisciplinar em saúde.

Embora existam no Brasil algumas escalas e questionários que facilitam a elaboração de diagnósticos no contexto familiar, orientando o desenho de intervenções, tais como *Family Health Behavior Scale*^7^*, Escala de Vulnerabilidade de Coelho* e *Savassi*^5^ e *Escala de Performance Paliativa*^6^, elas estão focadas na identificação de iniquidades e organização do serviço, e não necessariamente no estudo multidimensional que engloba os aspectos socioculturais e históricos da imagem familiar. Entender completamente a saúde de uma família é considerar fatores de funcionamento familiar, comunicação, resolução de problemas, *coping*, saúde mental, suporte emocional e econômico, comportamentos saudáveis e cuidados adequados, ou seja, temas transversais, aqui explorados de modo breve.

Conforme avaliado por meio do coeficiente de Kendall, houve uma concordância muito boa (0,84) entre as avaliações dos juízes, além do IVC predominante de 1 e um E-IVC de 0,98. Isso representa uma significativa concordância entre os especialistas em relação aos itens e ao instrumento. Em sua versão original, as duas versões da escala (curta e longa) demonstraram boa validade e confiabilidade para avaliar a saúde da família em serviços de cuidados primários ^8^. Todavia, as análises de confiabilidade e evidências de validade têm sido recomendadas pelos autores do instrumento como forma de verificar sua precisão e mensuração correta. Em nosso estudo, os itens obtidos pela confiabilidade interna das questões da versão final da escala foram satisfatórios e demonstram a validade da versão brasileira da *Family Health Scale*, mostrando que ela é precisa, reprodutível e consistente, o que foi confirmado pelos valores de coeficiente α de Cronbach, correlação item-total e coeficiente ω de McDonald. Ademais, o instrumento demonstrou evidências de validade convergente.

O único estudo que observou evidências de validade da *Family Health Scale* fora do cenário estadunidense ocorreu, recentemente, na China ^9^. Nessa pesquisa, observou-se que o α de Cronbach da versão chinesa foi de 0,83 para a versão curta e variou de 0,7 a 0,9 em relação aos quatro fatores da escala. Ademais, os conceitos de saúde da família traduzidos na escala não diferem muito do estudo original e podem ser utilizados na população brasileira.

A maioria dos itens da *Family Health Scale* foi invariante em relação a sexo, idade e estado civil ^8^, embora houvesse evidência de diferencial uniforme de alguns dos itens da escala com base nessas variáveis. Com relação ao sexo, os próprios autores da versão original da escala examinaram as propriedades psicométricas das versões longa e curta do instrumento entre casais e verificaram que tanto homens quanto mulheres respondem de forma semelhante sobre a saúde das suas famílias ^34^. Ainda, uma análise de sensibilidade foi realizada para verificar se variáveis socioeconômicas (estado civil, ter filhos, educação, idade, cor de pele e emprego) influenciavam no relacionamento familiar e a resposta foi negativa ^8^. Futuramente, é recomendável que sejam testados os fatores associados e/ou de predição para a saúde familiar excelente em diferentes regiões do Brasil.

Portanto, a *Family Health Scale* mostra-se como um recurso adequado e válido para que pesquisadores e profissionais da saúde consigam mensurar a saúde da família, suas tendências e interseções individuais, familiares e comunitárias, e possam, assim, medir fatores interdisciplinares essenciais para captar a dinâmica de uma família. Ademais, essa é uma escala que pode ser autoaplicável, ainda que sua interpretação seja útil para profissionais que desejem complementar suas ações em saúde.

Uma limitação deste estudo é a necessidade da realização de outras investigações para obtenção de mais evidências de validade e confiabilidade da escala, usando outras amostras representativas, considerando as diferentes regiões do Brasil. Outrossim, sugere-se a realização de pesquisas longitudinais, as quais seriam úteis para avaliação do questionário quanto à sensibilidade à mudança.

## Conclusão

A *Family Health Scale*, versão para língua portuguesa do Brasil, apresenta equivalência conceitual, semântica, cultural e operacional em relação aos itens originais, além de propriedades psicométricas satisfatórias para a aplicação direcionada à população brasileira.
